# Screening of minor psychiatric disorders and burnout among a sample of medical students in St. Petersburg, Russia: a descriptive study

**DOI:** 10.1186/s43045-021-00118-4

**Published:** 2021-08-26

**Authors:** Egor Chumakov, Nataliia Petrova, Tamila Mamatkhodjaeva, Antonio Ventriglio, Dinesh Bhugra, Andrew Molodynski

**Affiliations:** 1 St. Petersburg State University, 7/9 Universitetskaya Emb, Saint Petersburg, Russian Federation 199034; 2 St. Petersburg Psychiatric Hospital № 1 named after P.P. Kaschenko, 12 Kanonerskaya Street, Saint Petersburg, Russian Federation, 190121 Saint-Petersburg, Russia; 3 Department of Clinical and Experimental Medicine, University of Foggia, Foggia, Italy; 4 Institute of Psychiatry, King’s College London, London, UK; 5 Oxford Health NHS Foundation Trust, Oxford, UK; 6 Oxford University, Oxford, UK

**Keywords:** Medical students, Wellbeing, Professional burnout, Alcohol abuse, Stress

## Abstract

**Background:**

Despite the general interest of researchers around the world, there are few studies on the psychological wellbeing and burnout among medical students in Russia. The aim of this study was to perform screening for minor psychiatric disorders, burnout, problematic alcohol use, and quantify the psychological issues and stress among a sample of medical students in St. Petersburg, Russia.

**Results:**

According to the GHQ-12, screening for minor mental disorders was positive in 140 students (85%). Screening for burnout using the OLBI showed positive results in 121 (73%) students for disengagement and 132 (80%) students for exhaustion. Screening with the CAGE tool identified a risk of alcohol consumption in 33 students (20%). Most students reported academic studies as the main source of stress in their life (n = 147; 89.1%).

**Conclusions:**

This study identified very high levels of stress, burnout, risk of minor mental disorders, and problematic alcohol use among medical students in St. Petersburg, Russia. These findings suggest more attention is needed to the poor mental wellbeing and health in medical students in Russia.

## Background

The psychological adaptation of medical students and young doctors is of interest in many countries [1–6]. Research consistently shows a high prevalence of mental disorders and psychological stress among medical students [3, 7–9], significantly higher than in the general population [10, 11]. The incidence of depression and anxiety in a sample of Russian medical students was 4 and 6 times higher, respectively, than in students of other disciplines [12]. Also, of particular concern is the increasing prevalence of suicidal ideation in medical students [6, 9, 13, 14].

Medical students report pressure from their professional environment and academic studies are the main source of stress [3, 5, 15]. These stressful conditions may lead to high burnout rates [16–21]. Other sources of stress include psychosocial issues and environmental stressors [15, 22], financial problems, housing, and relationships [3]. The consequences of psychological adaptation difficulties in students are also shown in social life, leading to substance misuse [12, 23] and reduced learning achievements [24, 25].

The medical-training period is recognized as a crucial phase for the onset of mental disorders among doctors [10]. It is therefore relevant to study this phase as well as to develop prevention and psychohygiene strategies. Medical-training currently requires significant emotional and financial investment, so it is critical that faculty and managers provide help and support to these future physicians [9]. Mental health promotion for medical students calls for evidence-based interventions and psychosocial support [4]. Researchers strongly recommend widespread screening for symptoms of burnout and mental disorders in medical students in order to provide timely and appropriate interventions [6]. Despite the general interest of researchers around the world, there are few studies on psychological wellbeing and burnout among medical students in Russia [12, 22].

The aim of this study was to perform screening for minor psychiatric disorders, burnout, problematic alcohol use, and quantify the psychological issues and stress among a sample of medical students in St. Petersburg, Russia.

## Methods

### Procedure

An anonymous online survey of medical students of St. Petersburg State University Medical Faculty was conducted in May–June 2020. The research employed the online platform *questionpro.com*. The invitation to participate in the research has been sent through the mailing list of students’ council of the faculty and was also published in social networks groups for students. Reminders were sent at second and fourth week from the launch of the survey. Each student could complete the survey just one time and participation was anonymous and voluntary. Data were password-protected and answers were confidentially treated. Individual respondents could not be identified. The authors assert that all procedures contributing to this work comply with the ethical standards of the relevant national and institutional committee on human experimentation with the Helsinki Declaration of 1975, as revised in 2008. The study protocol was approved by the ethics committee of the St. Petersburg State University.

### Participants

A total of 174 students completed the survey (response rate 43.2%). We calculated that the sample size 161 or more respondents are needed to have a confidence level of 95% that the real value is within ± 6% of the surveyed value (the margin of error is 0.06). Since we expected that there might be difficulties in recruiting respondents for the online survey, we took into account a possible minor downward deviation from the common target values. Only nine students did not answer all the key questions in the survey, and these students were excluded from the final analysis. The final sample included 165 respondents who answered all the key questions of this survey.

### Measures

The survey was conducted in Russian and collected basic demographic information and structured questionnaires with proposed answers and the item “other” in those cases when the questions were not quantitative. Choosing “other” allowed respondents to provide their personalized answer. The first part of the survey consisted of a structured questionnaire that included questions about students’ mental health and psychological wellbeing before and during their studies at the university, including any history of mental health problems, use of prescription (and non-prescription) medications, drug and alcohol use, main sources of stress experienced by students, etc. The full text of the questionnaire is available from the first author upon request.

Minor psychiatric disorders were identified through the Short General Health Questionnaire (GHQ-12) [26]; Oldenburg Burnout Inventory (OLBI) was used to identify burnout [27], while problem alcohol use was identified using the CAGE (cut-down; annoyed; guilty; eye-opener) questionnaire [28]. For the GHQ-12, the bimodal GHQ scoring method (0-0-1-1) was used, and a score of two was considered as the cut-off to identify cases, based on standardized and validated results [29]. For the OLBI, burnout was detected by combining the mean score of 2.25 for *exhaustion* and 2.10 for *disengagement* [30]. Finally, for the CAGE questionnaire, a score of ≥ 2 was considered as the cut-off for problem drinking [31]. All ratings were previously translated and adapted in Russian: GHQ-12 Cronbach’s α = 0.751 [32]; OLBI disengagement Cronbach’s α = 0.652 [33]; OLBI Exhaustion Cronbach’s α = 0.838 [33]; CAGE Cronbach’s α = 0.71 [34].

### Statistical analysis

Data were entered and analyzed using IBM SPSS Statistics (Version 24). Research data are presented as the arithmetic mean and standard deviation (M ± SD). We used chi-square (*χ*^2^) tests for categorical variables and to compare proportions. The correlation between the indices was studied by means of a linear correlation analysis, the Pearson test. Correlation coefficient (*r*_s_) from 0.3 to 0.7 means a moderate positive; negative *r*_s_ corresponds to inverse correlation.

## Results

Sociodemographic characteristics of respondents are shown in Table 1. No students selected the item “others” in the question about gender. Most participants were in the 5th year of study, which may reflect the fact that these students were trained in “psychiatry, medical psychology”. Information regarding the educational achievements of parents was obtained from each student, with 1 (0.6%) indicating high school or below, 8 (4.8%) indicating GCSE (General Certificate of Secondary Education), 13 (7.9%) indicating A-Level or equivalent, 117 (70.9%) indicating undergraduate, and 26 (15.8%) indicating postgraduate education.

**Table 1:** Sociodemographic characteristics of 165 surveyed students

*Demographics*	*Number of students (%)*
*Gender*	
Female	132 (80.0%)
Male	33 (20.0%)
*Year of study*	
1st	28 (17.0%)
2nd	24 (14.5%)
3rd	29 (17.6%)
4th	19 (11.5%)
5th	39 (23.6%)
6th	26 (15.8%)

Assessing students’ mental health in the period before entering the university, 27 (16.4%) reported that during that period they had visited a general practitioner, psychologist, psychiatrist, psychotherapist, or other specialist in the field of mental health for reasons related to psychological issues (including reduced mood, anxiety, eating disorders, or obsessions). Ten individuals (6.1%) reported they had been diagnosed with mental health disorders before entering medical school, of which four students (2.4%) received an attention deficit hyperactivity disorder (ADHD) or autism spectrum disorder diagnosis. 10.3% of students (n = 17) also indicated that they had been prescribed medications for a mental disorder (including depression, anxiety, psychosis, ADHD) during that period.

Twenty-five students (15.2%) reported they had been diagnosed with a mental disorder while at university whereas 18 students (10.9%) indicated that they were having care by a general practitioner, psychologist, psychiatrist, psychotherapist, or other mental health professional during their participation in the study. The same number of students (n = 18; 10.9%) reported they were on a maintenance treatment during the survey.

Students reported academic studies as the main source of stress in their life (n = 147; 89.1%). Other sources of stress included social relationships (intimate or family; n = 84; 50.9%), financial wellbeing (n = 63; 38.2%), work (n = 53; 32.1%), and housing problems (n = 34; 20.6%). Ten students (6.1%) also identified other sources of stress such as low self-esteem, social problems, anxiety about their own health or health of their relatives, career after graduation, and existential issues. Most respondents reported having two (n = 60; 36.4%) or three (n = 43; 26.1%) main sources of stress. One source of stress was reported by 30 (18.2%) respondents, while four and five sources were reported by 15 (9.1%) and 10 (6.1%) students, respectively. Only seven people (4.2%) did not report any stress in their lives.

Screening with the CAGE tool identified alcohol problems in 33 students (20.0%). 23.6% of students (n = 39) had experience with illegal drugs, including cannabis (n = 37; 22.4%), ecstasy (n = 10; 6.1%), amphetamines (n = 7; 4.2%), cocaine (n = 2; 1.2%), opiates (n = 2; 1.2%), and others (n = 8; 4.8%). The number of students who were worried about psychoactive substance use was very low (n = 5; 3.6%). Only 4.8% (n = 8) of respondents indicated that others expressed concerns about their own substance use.

More than one-third of respondents (n = 63; 38.3%) reported that they had taken a psychoactive substance in the last year before the course in order to improve their concentration or academic performance (excluding caffeine or other energy drinks). Forty-five students (27.3%) reported taking non-prescription substance or medications outside their intended use to feel better or uplift their mood.

According to the GHQ-12, 140 students (84.8%) had a total score of 2 or higher, indicating a high risk of minor mental disorders in the sample. The mean value of the total GHQ-12 score in the study group was 5.05 ± 3.04. The screening for burnout using the OLBI showed positive scores in 121 (73.3%) students for disengagement and 132 (80.0%) students for exhaustion. A positive statistically significant correlation has been found between the overall GHQ-12 score and OLBI disengagement and exhaustion scores (Table 2), as well as between individual OLBI scores. The correlation between training course and OLBI disengagement was less than moderate. No differences were found in the frequency of positive screening of the survey’s techniques when dividing respondents by gender (Table 3).

**Table 2:** Correlations between the studied values

		Year of study	CAGE	GHQ-12	Disegagement	Exhaustion
Year of study	r		− .078	− .082	.192^*^	.139
	p		.321	.296	.014	.076
CAGE	r	− .078		.083	.171^*^	.143
	p	.321		.289	.028	.066
GHQ12	r	− .082	.083		.442^**^	.516^**^
	p	.296	.289		.000	.000
Disegagement	r	.192^*^	.171^*^	.442^**^		.750^**^
	p	.014	.028	.000		.000
Exhaustion	r	.139	.143	.516^**^	.750^**^	
	p	.076	.066	.000	.000	

*r* Pearson coefficient, *p* statistical significance

*p < 0.05

**p < 0.001

**Table 3:** Rates of positive GHQ-12, OLBI disengagement, OLBI exhaustion, and CAGE screening by gender of respondents

Screening techniques	Female students (n = 132)	Male students (n = 33)	*χ*^2^ (df), *p*
	n (%)	n (%)	
GHQ-12	112 (84.8%)	28 (84.8%)	*χ*^2^(1) = 0, *p* = 1.0
OLBI			
Disegagement	100 (75.8%)	21 (63.6%)	*χ*^2^(1) = 1.98, *p* = 0.159
Exhaustion	108 (81.8%)	24 (72.7%)	*χ*^2^(1) = 1.36, *p* = 0.24
CAGE	26 (19.7%)	7 (21.2%)	*χ*^2^(1) = 0.038, *p* = 0.85

## Discussion

This survey collected further and relevant evidence regarding the wellbeing and health issues of medical students in St. Petersburg, Russia. Young people’s health has a potential impact on future population health and global economic development unless timely and effective strategies are adopted [35].

Some differences in the frequency of mental disorders were found in respondents who reported being diagnosed before entering university (6.1%) or during their own training (15.2%), while GHQ-12 screening for general (non-psychotic) mental health problems was positive in 84.8% of respondents. In a previous study conducted in Russia, clinically significant symptoms of social phobia and generalized anxiety were found in 16% of medical students, while symptoms of depression (according to the depression anxiety stress scale-21) were observed in 34% of medical students [12]. The frequency (27.3%) of use of non-prescription substance or medications outside prescription over the past year is alarming, indicating a high probability of self-treatment in the study group. Although a screening survey is not sufficient to make a diagnosis, data obtained clearly indicated a high probability of common mental disorders in the study sample.

Academic stress and the pressure of the professional environment were rated as the leading global sources of stress in medical students [3, 5, 15]. This was confirmed in our survey, where academic studies were the most frequently cited source of stress (89% of respondents). Further evidence of the importance of academic stress among our respondents was the widespread taking of medications aimed at increasing concentration or improving academic performance (38.3%) over the past year. In another study from Russia, 26.0%, 69.1%, and 4.9% medical students reported low, moderate, and high perceived stress respectively [22]. According to the literature, perceived stress in medical students was higher among older groups and final year medical students [15], but this was not confirmed in our study.

There is no doubt that study or work places affect our mental health and wellbeing [1]. The burnout of health workers is an important contributory factor in medical errors and reduced quality of medical care [7]. Thus, it is very important to focus on burnout prevention during the training period. Our study found high frequencies of both disengagement (73.3%) and exhaustion (80.0%). The relationship found in the study between the frequency of burnout symptoms and the GHQ-12 score appears to confirm the potential link between burnout and the risk of developing mental disorders, particularly depression [36]. These indicators are discouraging and should be treated as a call for direct action to improve the psychological wellbeing of students. It should be noted, however, that the reported frequency of emotional burnout symptoms among Russian medical students is lower than in many other countries [3]. Further studies are required to assess the possible causes of these cultural differences, as well as the socio-cultural factors potentially associated with them.

One in five students has shown signs of alcohol problems using the CAGE questionnaire, significantly higher than in other countries [3]. Our data are consistent with literature from Russia. In fact, in a previous study on alcohol use, heavy drinking, and problem behavior among Russian Federation university students, heavy alcohol use was revealed in 20.4% of them [37]. Another study found that heavy drinking among university students in Russia was common for 37.1% of men and 39.6% of women [38], and those were the highest rates among 24 countries. Alcohol use was the leading risk factor for death among young people aged 15–19 and 20–24 in both 1990 and 2013 Global Burden of Disease Study reports [35]. However, a recent study showed a clear trend toward a decline in alcohol consumption among adolescents and young adults under 25 in Russia [39], which may explain the differences in frequencies between our and previous studies.

In order to determine potential risk groups for burnout, problematic alcohol use and risk of general (non-psychotic) mental health problems, the results of the study were compared according to participants’ gender. No statistically significant differences were found in the studied items. Moreover, no statistically significant correlation between the studied indicators and the students’ course of study was obtained. The reason for this may be the relatively small sample in the study.

This study was conducted during the period of social restrictions imposed in St. Petersburg to combat the spreading of COVID-19. Although there was no official ban on leaving home, movement around the city and students’ social contacts were significantly limited and all university classes were converted to remote learning. Not surprisingly, research in Russia over the past year has confirmed that during the COVID-19 pandemic lockdown has led to emotional disturbance, depression, irritability, insomnia, anger, and emotional exhaustion among other things [40, 41]. Young people have been particularly exposed to psychological stress during the period of social isolation in Russia [41]. Although we did not assess the direct association between the results of the study and the finding of respondents in self-isolation, the authors report that at the time of the study all students participating in the study were at least switched to distance learning and were subject to general instructions from the St. Petersburg and Russian governments with recommendations for self-isolation. These additional external factors may have affected the level of stress and burnout in the study sample, so it may be advisable to compare our findings with further studies after the end of pandemic.

In summary, this research sheds some light on the problem of psychological wellbeing and health of medical students in Russia. Academic schedules and load of medical students should be balanced to prevent educational stress, anxiety, and depression [12]. Administrative measures should focus on developing preventative strategies for stress management to improve students’ psychological wellbeing [22]. We also hope that our study motivated the participating medical students to self-reflect and try to optimize their psychological state.

### Strengths and limitations

The main strength of this research was the employment of reliable screening tools, extensively and internationally used as in some previous studies on the psychological wellbeing of medical students. Despite its originality, this study has some limitations.

It was based on an online survey which guaranteed confidentiality, but respondents were self-selected and theoretically it is possible that those who were experiencing problems may have been more likely to respond. Diagnoses of mental disorders are also self-reported and not clinically confirmed. The study was conducted during the COVID-19 pandemic quarantine measures and lockdown, which may have affected stress levels, burnout, and current mental health problems in the sample. Therefore, it would be useful to conduct the study in dynamics after the removal of all social restrictions. Also, it would have been interesting and useful to conduct a comparison with students from other disciplines such as psychology, social workers students, and dentistry students.

## Conclusion

This study reported high levels of burnout, stress, problematic alcohol use, and risk of minor mental disorders in medical students in St. Petersburg, Russia. It may suggest more attention to the mental wellbeing and health in medical students in Russia. Also, these findings might suggest strategies to improve mental health, contrast stigma, and discrimination, and prevent mental disorders among medical students.

## Data Availability

The datasets used and/or analyzed during the current study are available from the corresponding author on reasonable request.

## Abbreviations

GHQ-12: Short General Health Questionnaire
OLBI: Oldenburg Burnout Inventory
CAGE: Cut-down, Annoyed, Guilty, Eye-opener
M: Mean
SD: Standard deviation
GCSE: General Certificate of Secondary Education
ADHD: Attention deficit hyperactivity disorder
